# Low-cost photocatalytic membrane modified with green heterojunction TiO_2_/ZnO nanoparticles prepared from waste

**DOI:** 10.1038/s41598-023-49516-0

**Published:** 2023-12-13

**Authors:** Sahar A. Mousa, Heba Abdallah, S. A. Khairy

**Affiliations:** 1 Physics Department, Faculty of Science, Cairo University, Giza, 12613 Egypt; 2 Chemical Engineering and Pilot Plant Department, Engineering Research Division, National Research Centre, 33 El-Bohouth St. (Former El-Tahrir St.), Dokki, PO Box 12622, Giza, Egypt

**Keywords:** Materials science, Physics

## Abstract

The combination of photocatalysis and membrane procedures represents a promising approach for water treatment. This study utilized green synthesis methods to produce TiO_2_ nanoparticles (NPs) using Pomegranate extract and ZnO nanoparticles using Tangerine extract. These nanoparticles were then incorporated into a polyvinyl chloride (PVC) nanocomposite photocatalytic membrane. Different devices were used to examine the properties of nanocomposite membranes. The prepared membranes' morphology was examined using atomic force microscopy (AFM) and field emission scanning electron microscopy (FESEM). The hydrophilicity of the membrane surface was assessed through the measurement of contact angle, while the crystal structure and chemical bonding were analyzed using Raman and Fourier transform infrared spectroscopy (FT-IR). The study also encompassed an examination of the mechanical properties. The hydrophilicity of the modified membrane exhibited a significant improvement. Additionally, there was an observed increase in both the pure water flux and rejection values. The photocatalytic activity of the membrane was found to be enhanced when exposed to sunlight as compared to when kept in the dark. The TiO_2_/ZnO nanocomposites membrane exhibited the highest level of photocatalytic degradation, achieving a rejection rate of 98.7% compared to the unmodified membrane. Therefore, it was determined that the TiO_2_/ZnO nanocomposites membrane exhibited superior performance to the other membranes assessed. The potential utility of our research lies in its application within the water treatment industry, specifically as an effective technique for modifying PVC membranes.

## Introduction

Membrane technology has emerged as a promising method for separation due to its cost-effectiveness and excellent separation performance. Membrane separation technologies are extensively employed in various industries such as biotechnology, dairy, food, and water treatment due to their notable efficacy, functional apparatus, and low energy consumption. The fouling phenomenon poses a significant limitation in the application of membranes. During filtration, the membrane surface experiences the adhesion of colloidal particles, macromolecules, proteins, and various other substances, resulting in the phenomenon known as membrane fouling. The reduction in permeation flux, shelf life, and separation ability of a membrane is ultimately caused by fouling, which occurs due to changing the membrane selectivity^1–3^.

Poly (vinyl chloride), commonly referred to as PVC, is widely recognized as a highly preferred membrane material due to its affordability, resistance to acid, alkali, and microbial agents, as well as its commendable mechanical strength. Unfortunately, PVC membranes exhibit a restricted scope of applications due to their inherent low flux and susceptibility to fouling. Considerable endeavors are currently being undertaken to enhance these membranes’ performance and antifouling characteristics. One of the most prevalent techniques utilized for enhancing or refining is the process of blending in the production of PVC-based membranes, wherein hydrophilic or amphiphilic additives are incorporated into the dope solution before casting. The blending process is easy and dependable^4–11^. However, the regulation of membrane fouling is still limited in terms of external functionalities, such as operational conditions, due to the complexity and variety of feed fluids. There has been an increasing interest in self-cleaning or functional membranes that can catalyze foulant-degrading processes or decompose foulants on the membrane surface. The simultaneous filtration and degradation of organic pollutants can be achieved by fusing the membrane substance with photocatalytic activity, resulting in a photocatalytic membrane (PMR)^12^.

Photocatalysis is a cost-effective and durable method that rapidly converts organic pollutants into inorganic small molecules, such as H_2_O and CO_2_. Unfortunately, several challenges continue to exist, such as the separation of catalysts and various other issues. In this case, the combination of membrane separation and photocatalysis with photocatalytic-membrane coupling preserves the initial benefits of these technologies while also resolving and minimizing the issues that have impeded their development^13–15^. The TiO_2_ photocatalyst is extensively utilized in the domain of water treatment due to its affordability, non-toxic nature, remarkable photochemical stability, and absence of secondary pollutants^16,17^. Water filtering membranes are improved in flux, pollutant removal, and contamination resistance when TiO_2_ NPs are introduced^18^. In the presence of sunlight, a photocatalytic membrane composed solely of TiO_2_ exhibits relatively low levels of photocatalytic activity^19^. This issue has significantly hindered the utilization of TiO_2_ as a photocatalyst. Therefore, it is imperative to propose a feasible and cost-effective approach for mitigating membrane fouling^20^. A large amount of energy is needed to remove membrane fouling, which reduces process efficiency and raises costs. Moreover, routine membrane chemical cleaning pairs performance and shortens its lifespan^21^.

Some scientists have investigated embedding inorganic nanoparticles like SiO_2_^8^, ZnO^22^, and TiO_2_^23^ into PVC membranes. The polymeric membrane matrix's chemical, physical, and mechanical characteristics, pore formation, surface hydrophilicity, porosity, and antifouling capabilities were all improved by adding TiO_2_ nanoparticles^20,24^. ZnO's superior electron transport capabilities are believed to be perfect for extending the TiO_2_ photoresponse range^25^. The lifetime of electron–hole pairs can be significantly extended by using ZnO as an electron transporter for TiO_2_ NPs^26^.

The synthetic methods utilized in the production of nanoparticles (NPs) are characterized by high costs and adverse environmental impacts. Consequently, the utilization of plant-assisted, greener nanomaterials is deemed more favorable compared to chemical processes. To mitigate the utilization of deleterious reagents in the synthesis process, the implementation of biosynthesis serves as a highly effective approach to produce NPs by employing cost-effective, environmentally friendly, and biologically derived precursors^26^. The biosynthesis process typically uses three different types of biological precursors, such as bacteria, enzymes, and plant extracts^27^. The utilization of plant extracts to produce nanoparticles is considered a highly viable approach due to the abundance and cost-effectiveness of these extracts. Moreover, plant-based NP synthesis techniques are simple to scale up and are frequently chosen because of their environmentally beneficial approach. Plant metabolites, such as alkaloids and phenolic chemicals, exhibit effective reducing properties.

Polyethersulfone (PES)^28^, polysulfone (PSF)^29^, polyvinylidene fluoride (PVDF)^30–32^, polyacrylonitrile (PAN)^16,33^, polyvinyl chloride (PVC)^17,34^, and cellulose acetate (CA)^18,35^ have all been used to prepare membranes for photocatalytic membranes using the phase inversion method.

In the present work, PVC membranes modified with green synthesized TiO_2_, ZnO NPs, and TiO_2_/ZnO nanocomposite membranes are manufactured and used for wastewater treatment. To reduce the thickness of the cake layer on the surface of the membrane, we will exploit the hydrophilic properties of TiO_2_ NPs to achieve this purpose in addition to increasing the flux of the system. Nanocomposites composed of TiO_2_/ZnO were used to investigate their ability to reduce the recombination process and increase carrier lifetime. All these investigations were carried out to increase the rejection and water flux through the membrane.

## Materials and methods

### Materials

Poly (vinyl chloride) (PVC, high molecular weight), polyvinylpyrrolidone (K25) N, and N-dimethylacetamide (DMAc, 99%) were obtained from Qualikem Fine Chem Pvt. Ltd. (India). Humic acid (60%) (Loba), titanium tetrachloride (TiCl_4_), Zinc acetate dehydrate (CH_3_COO)_2_Zn.2H_2_O (98% purity) supplied by Loba Chemie (India), Pomegranate peels, orange peels from Egyptian local market.

### Collection of plant material

The collection of Orange and pomegranate (plants) material was performed according to institutional, national, and international guidelines. Plant studies and all experimental procedures were performed per applicable institutional, national, and international guidelines. Pomegranate was obtained from Egypt's local market and Orange from farms in upper Egypt. No certification or permission was needed to collect the samples because the questioned species is extensively spread nationwide. However, our search from the IUCN database found that Pomegranate and Orange are not red-listed or classified as a threatened species.

### Preparation of NPS

#### Orange extract

To remove dust, 10 g of orange peels were washed several times under running water. These leaves were washed several times with DW and dried at 50 ºC. The dried orange peels were boiled in 100 ml of DW until a yellow-colored solution formed at 100 °C 2 h and then cooled to room temperature. Following cooling, the extract was filtered through Wattman filter papers to obtain the desired extract.

#### Pomegranate extract

The pomegranates were repeatedly washed under running water to remove dust. After that, DW was used multiple times to wash the pomegranate. Fresh pomegranate peels weighing 240 g were cut into small pieces, heated in 1200 mL DW until boiling, and then cooled at room temperature. After cooling, the extract was filtered through Wattman filter papers to obtain the necessary extract.

#### Preparation of ZnO NPs

After stirring 600 ml of orange peel extract for 30 min, gradually add 12 g zinc acetate. Under identical conditions, the reaction was maintained for 30 min. The temperature then rose to 80 °C. The solution was kept in this state for 2 h. The particle was washed several times with DW and dried at 80 °C. At 450 °C, it was then calcined for 3 h.

#### Preparation of TiO_2_ NPs

A volume of 500 mL of pomegranate extract was subjected to agitation for 30 min, followed by refrigeration. The beaker, which held the plant extract, was incrementally filled with a moderate quantity of TiCl_4_. The reaction was submerged in an ice bath for a full hour. Subsequently, the temperature was raised to 80 °C and maintained at this level for two hours. Before drying at 70 °C, the TiO_2_ NPs were washed several times with DW. The powders were then calcined for three hours at 450 °C^30^.

### Preparation of photocatalytic nanocomposite membranes

**Figure 1:**
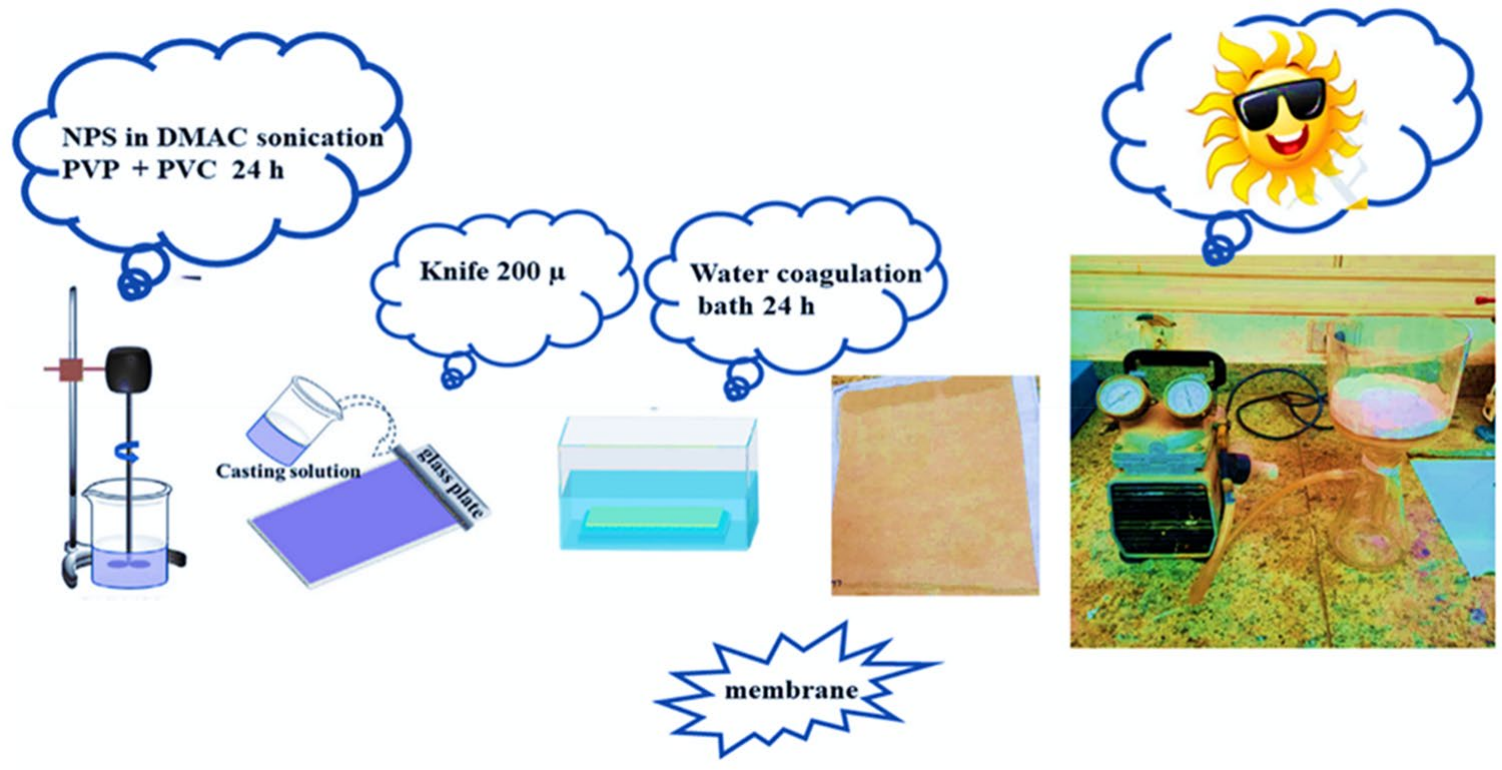
Preparation procedure of photocatalytic nanocomposite membranes.

Photocatalytic flat sheet membranes were created Using phase inversion. The preparation procedure is depicted in Fig. 1; 2 g of NPs were initially ultrasonically dispersed for 1 h in 84 g of DMAc. After adding 2 g of PVP, 12 g of PVC was dissolved in the solution with vigorous stirring to produce a homogenous casting solution. Subsequently, the degassed mixture is poured onto a glass plate, utilizing a casting knife with a thickness of 200 µm, which is carefully drawn across a non-woven fabric. To complete the solvent/nonsolvent separation, the cast films were immersed in a coagulation bath of distilled water without the solvent evaporating. The prepared flat sheet membrane was thoroughly coagulated, rinsed multiple times in DW to remove residual solvent, and stored in clean water for additional testing.

### Characterization of photocatalysts and membrane

#### Photocatalyst characteristics

A D8-Advance Bruker AXS diffractometer was used to identify the structure and crystallinity of the synthesized NPs. High-resolution transmission electron microscopy (HRTEM) was used to describe their morphology with an accelerating voltage of 100 kV, TEM-1230 (JEOL Co., Japan) performed transmission electron microscopy. The energy bandgap of NPS was determined using diffused reflectance UV–Vis spectroscopy.

#### Membrane characterization

##### Field emission Scanning electron microscope (FE-SEM)

Field emission scanning electron microscope, model JSM-T20 JEOL, Japan, was used to describe the cross-sectional, and surface morphologies of the prepared membranes.

##### The Surface roughness: topography and roughness

Using an atomic force microscope (AFM) (Agilent Technologies, 5600 LS), the topography and roughness of the top membrane surfaces were examined. On top of a circular sample holder measuring 1 cm^2^, a tiny portion of each membrane was cut and adhered.

A contact angle goniometer (Attention, Theta, by Biolin Scientific) outfitted with image-processing software was used to calculate the membrane's contact angle. This instrument was used to test how hydrophilic the additional NPs were.

FT-IR measurements of the chemical components of the manufactured membrane's surface were discovered using Fourier transform infrared spectroscopy (FT-IR, PE-100, US). All of the spectra were recorded in wavenumber between 400 and 4000 cm^−1^. with the samples mounted on a sample holder.

##### Raman analyses

A Confocal Raman Microscope (Witec, 300alpaR, made in Germany) was used to conduct Raman studies. A laser (785 nm) was used to excite the samples, and measurements were taken with the microscope's beam path adjusted to 50X.

##### Mechanical characterization

An electric elastic yarn strength analyzer was used to assess the membranes' mechanical characteristics, such as their tensile strength and elongation (YG020B, Nantong Sansi Co. Ltd., China). The measurements were made at a constant crosshead rate of 2 mm/min at room temperature.

##### Porosity, pore radius, and pure water flux of membranes

Porosity using the following equation Eq. (1)^36^, water uptake tests were used to calculate the overall porosity (%) of membranes as a function of membrane weight.

1 $${\varepsilon }\left( \% \right) = \frac{{{\text{m}}1 - {\text{m}}2}}{A\;d\;\rho } \times 100$$

where the weights of the membrane in its dry and wet conditions, are m_2_ and m_1_, respectively. A stand for the evaluated membrane sample’s effective area, d for the membrane's thickness, and *ρ* for pure water's density. The synthetic membranes were submerged in pure water for 24 h before the measurement to achieve the equilibrium swelling state.

the total volume of the porous membrane divided by the volume of the holes is known as the membrane porosity. The membrane will be weighed, dried in an oven for 24 h at 100 °C, soaked overnight in deionized water at room temperature, and wiped dry reweighing after wiping away any surface moisture with absorbent paper. The Guerout-Elford-Ferry equation (Eq. (2)): was used to get the mean membrane pore radius^37–40^.

2 $$rm = \sqrt[2]{{\frac{{\left( {2.9 - 1.7Q\varepsilon } \right) \times \eta LQ}}{ \varepsilon \times A \times \Delta P}}}$$

where L is the membrane thickness (m), Q is the permeate water volume per unit time (m^3^ s^−1^)), ε is the porosity, *η* is the water viscosity $(8.9\times10^{-4} {\text{Pa.s}})$, and ΔP is the load pressure (Pa). The generated membranes' water flux J $(\text{L.m}^{2}.{\text{h}}^{-1})$ was determined using Eq. (3)^41^.

3 $$J = \frac{V}{{A{ }\;\Delta { }t}}$$

where Δt is the time, A for the effective membrane area (m), and V for permeate per unit time (h).

#### Photocatalytic performance tests

The performance of the membranes as prepared for photocatalysis was tested using degradation systems with dark, and sunlight. For the dynamic system, the transmembrane pressure was fixed at 1.65 MPa, and the humic acid concentration in the feed water was 500 mg L^−1^, with PH = 9.5 and temperature 25 °C. Samples of the reaction solution collected at predetermined intervals during visible light irradiation. The humic acid concentration was measured using a Cary UV-2450 spectrophotometer at 254 nm^42^. The following equation describes the degradation efficiency of humic acid Eq. (4), represents the photocatalytic activity, Degradation of humic acid (η%), C0, and C are the concentration of humic acid before and after treatment Eq. (4)^43^:

4 $$\eta = \frac{C0 - C}{{C0}} \times 100\%$$

## Characterization of the fabricated membranes

### NPs characterization

#### XRD of the synthesized NPs and crystallite size.

**Figure 2:**
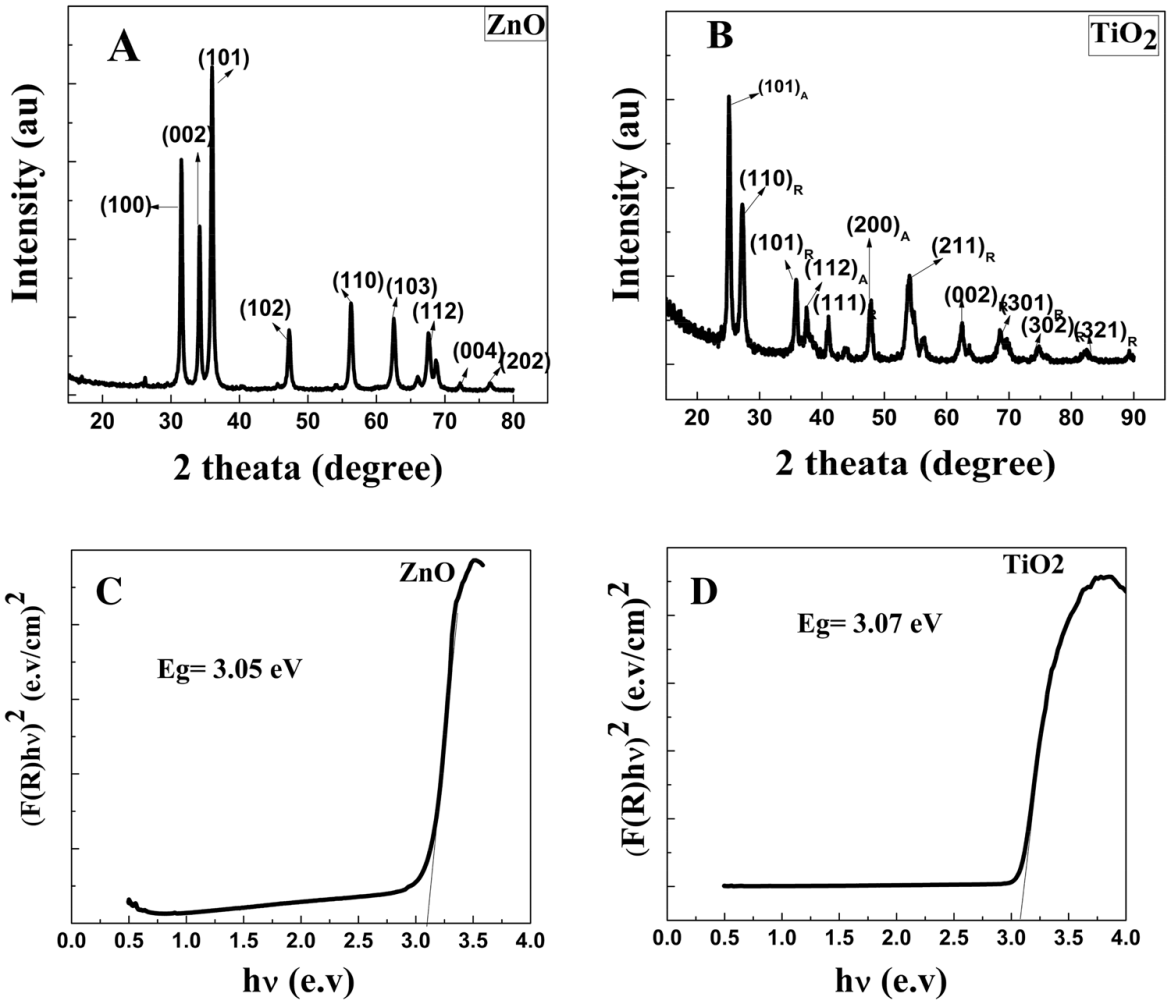
XRD pattern for (ZnO (**A**), and TiO_2_ (**B**)), and the plots of (αhν)^2^ vs hν for (ZnO (**C**), and TiO_2_ (**D**)).

The XRD configurations of the prepared TiO_2_, and ZnO NPs shown in Fig. 2A and B. The typical XRD configuration of the prepared TiO_2_ NPs demonstrated numerous well-defined diffraction reflections. The appearance of diffraction reflections at (101), (112), (200), (213), (116), and (215) with a tetragonal structure anatase phase [File Card No: 04-014-0493] and characteristic peaks at (110), (101), (112) (200), (211), (220), (002), (320), (301), and (310) with a tetragonal structure rutile phase [File Card No:04-012-7240] for TiO_2_ NPs. Furthermore, characteristic peaks were indicated at (100), (002), (101), (102), (110), (103), (200), (112), (201), (004), and (202) with a hexagonal structure Zincite phase [File Card No:01-089-1397]. The sharpness and strong diffraction reflections in the XRD peaks confirmed that the prepared NPs have good crystallinity. No characteristic reflection allied to the impurities was detected in the pattern. Using the Scherrer equation eq, the average crystal size was calculated from the highest diffraction peak intensity (5)^44^. It was 24 nm for TiO_2_ and 39.5 nm for ZnO.

5 $${\text{D}} = \frac{0.9.\lambda }{{\beta \;cos\theta }}$$

where the Scherer constant (0.9) is added to the equation when using half-height width, λ is the X-ray wavelength, and β is the half-height width of the most intense diffraction peak (rad), and ϴ is the Bragg diffraction angle (°). The microstrain (ε), and the dislocation density (δ) and are calculated using the following equations Eq. (6,7).

6 $$\varepsilon = \frac{\beta cos\theta }{4}$$

7 $$\delta = \frac{1}{D2}$$

The dislocation densities were 1.7, and 0.6 × 10^15^ m^2^ for TiO_2_, and ZnO NPs, where the microstrain was 0.0017, and 0.0006 for TiO_2_, and ZnO NPs. The small dislocation densities of the prepared NPs indicate higher crystallization of samples^45^. Two phases anatase and rutile were observed for TiO_2_ NPs although the calcination temperature was 450 °C. Because of the preparation of the TiO_2_ NPs photocatalyst using green extract as reducing agent was much faster than using the chemical method and rutile phase affected by the reaction time. Furthermore, the Rutile phase is also affected by the presence of metal ions, which came from plant extract during the preparation of the TiO_2_ NPs photocatalyst^46,47^.

#### Optical properties

The band gap energy of the NPs was estimated using the Kubelka–Munk function equation Eq. (8). The Kubelka–Munk function F(R), which may be used to investigate the particles, gives the diffused reflectance as a function of absorption coefficient (8)^48^.

8 $${\text{F}}\left( {\text{R}} \right) = \frac{{\left( {1 - R} \right)^{2} }}{2R}$$

where R: reflectance and F(R): Kubelka–Munk functions. Equation (9) from the Tauc relation was used to compute the optical energy gap^49^.

9 $$F\left( R \right)h\nu = A\left( {h\nu - Eg} \right)^{{{1}/{2}}}$$

Plotting the (F(R)hν)^1/2^ vs. the energy of absorbed light in an indirect transition semiconductor as illustrated in Fig. 2C and D allowed us to measure the band gap energy (Eg) of the constructed membrane. The optical Eg of the synthetic ZnO and TiO_2_ NPs were 3.06, and 3.07 (eV) respectively. the value of Eg is smaller than those obtained theoretically. The anionic or cationic vacancies, antisemites, and interstitial vacancies that are generated during experimental work (came from plant extracts) may shift the absorption spectra towards the visible region^46,50^. However, the main changes in the optical energy gap can be associated with structural defects or additional states localized inside the bandgap. These defects might result from two factors: changes in the surface area and distinct types of oxygen vacancies. An increase in the number of defects leads to the formation of localized states within the bandgap due to the corresponding increase of vacancies. This behavior is an indication that these samples present a certain structural disorder degree. The presence of these crystal defects in the prepared NPs, acting as recombination centers, could induce energy levels within the bandgap and narrow the bandgap^51,52^.

#### HRTEM, and SAED patterns

**Figure 3:**
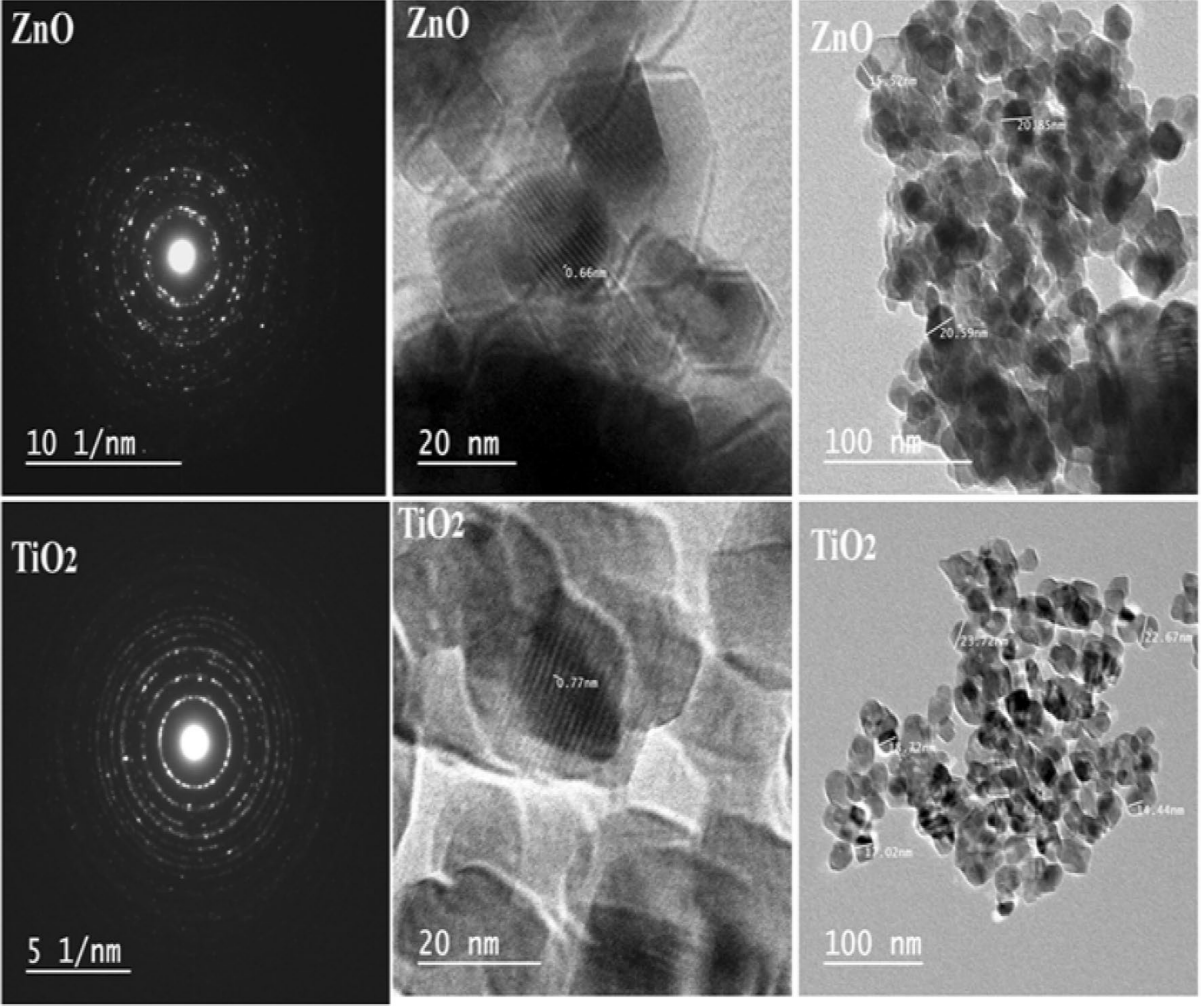
The HRTEM images, and SAED pattern for ZnO, and TiO_2_ NPs.

To acquire straight evidence about the shape in addition to the size of the photocatalyst NPs, HRTEM analyses were checked Examples of individual NPs under higher magnification showing lattice fringes a fringe type pattern is observed for ZnO, and TiO_2_ NPs Fig. 3). TEM images of TiO_2_ NPs showed that both are separated, dispersed, and homogenous in shape with nano-metric size range^46,53^. For ZnO the NPs have a hexagonal shape. The bright circular rings are demonstrated by the selected area electron diffraction (SAED) pattern., suggesting the highly nanocrystalline nature of TiO_2_ NPs^26,54,55^.

#### Membrane morphology, and the elemental analysis

**Figure 4:**
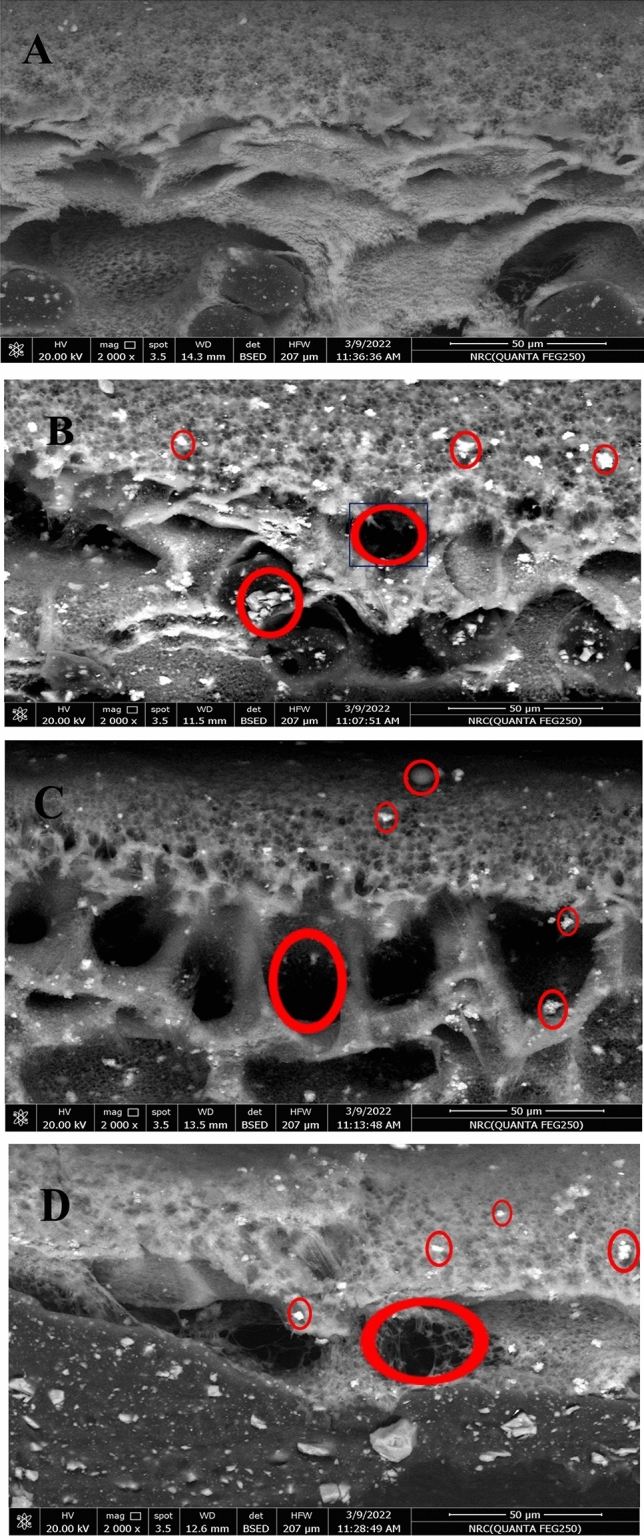
The cross-sectional FESEM images of the (blank, TiO_2_, ZnO, and TiO_2_/ZnO) membranes*.*

**The cross-sectional FESEM** images of the (blank, TiO_2_, ZnO, and TiO_2_/ZnO) membranes are displayed in Fig. 4 (A, B, C, and D), respectively. All membranes have an asymmetrical porous structure with a thick skin layer and soft finger-like sublayers. The system's kinetic and thermodynamic characteristics are changed by adding NPs to the PVC casting solution. The solvent (DMAc)-nonsolvent (water) exchange rate in the coagulation bath is enhanced, or water diffusion to the polymeric layer is improved because of the hydrophilic qualities of the NPs. Furthermore, no cracks were detected on the membrane surface, indicating that the incorporation of NPs did not cause the membranes to become brittle and had no detrimental effect on the membrane's strength. NPs are the white dots that appear on the surface of the membranes (within the red circle). There was no evidence of NP aggregation due to their low concentration and good dispersion in the polymeric membrane solution, indicating that the NPs were uniformly dispersed in the casting solution and membrane matrix^36,56–59^.

**Figure 5:**
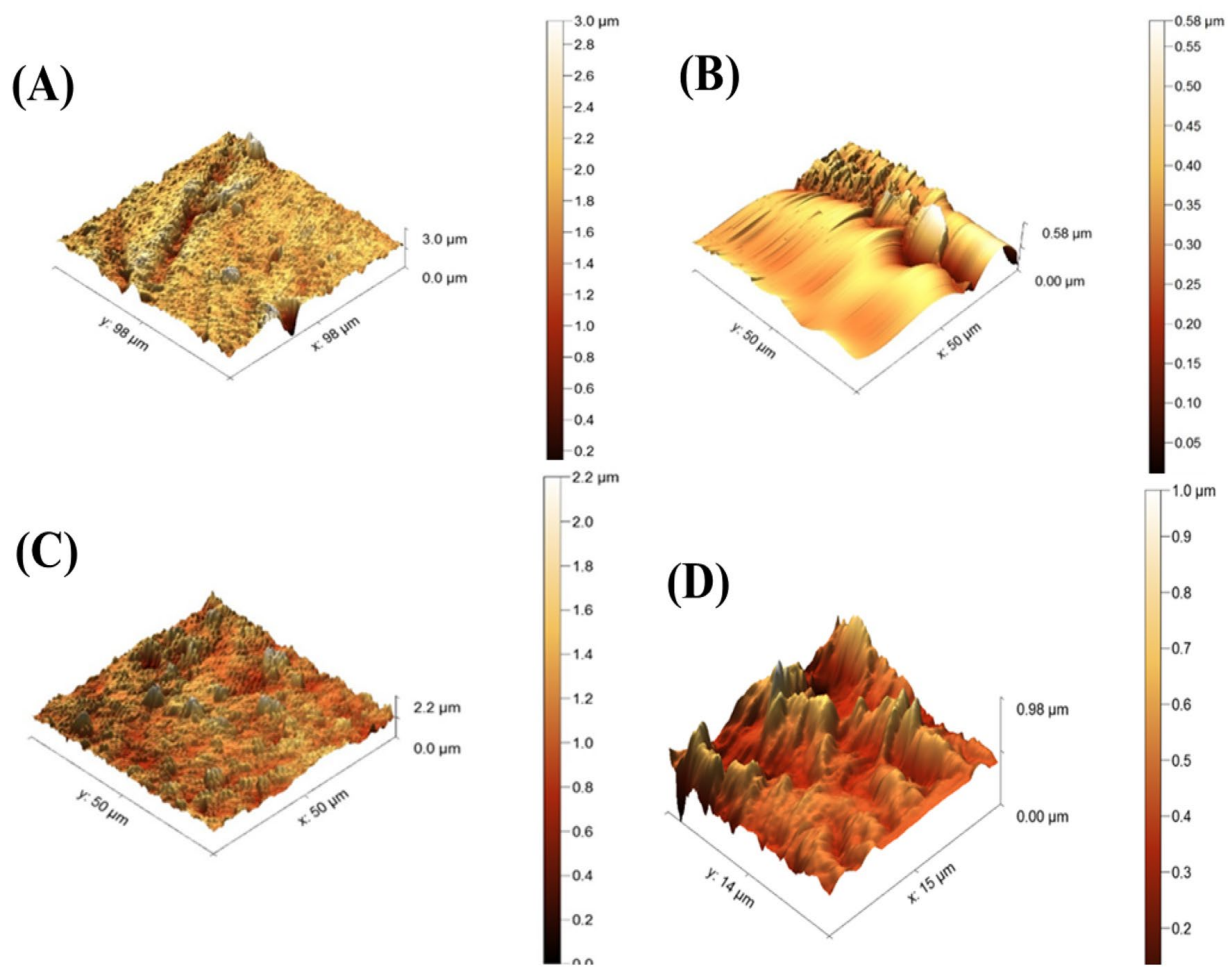
Three-dimensional AFM photographs of prepared membranes (blank, TiO_2_, ZnO, and ZnO/TiO_2_ nanocomposite membranes).

**AFM images** Fig. 5 demonstrates three-dimensional AFM pictures of manufactured membranes (blank, TiO_2_, ZnO, and ZnO/TiO_2_), as well as their surface roughness parameters. According to the AFM results in Table 1, the locations with the most brightness correspond to the membrane's highest peaks, while those with the most darkness correspond to its lowest valleys or pores. The roughness of the prepared membranes was 72.4, 78.3, 76.5, and 26.9 for (blank, TiO_2_, ZnO, and ZnO/TiO_2_), respectively, as depicted in Table 1. In general, hydrogen bonds between the NPs' functional groups (OH and –COOH) and the polymer may be responsible for hydrophilic NPs' impacts on the surface roughness of polymeric membranes. Reduced surface roughness decreases the likelihood that pollutants may aggregate in the membrane's surface troughs. Therefore, the antifouling properties of polymeric membranes are enhanced.

**Table 1:** Porosity, pore radius, roughness, contact angle, Rejection, and flux for (blank, TiO_2_, ZnO, and ZnO/TiO_2_) prepared membranes.

Sample	Porosity (%)	Rm (nm)	Ra (nm)	Contact angle degree	Rejection (%)	FluxL/m^2^ h
Blank	59 ± 0.3	260 ± 5	72.4 ± 0.2	69.0 ± 1.9	76.7 ± 5	1.5 ± 0.10
TiO_2_	60 ± 0.3	200 ± 5	78.3 ± 0.2	59.3 ± 1.6	90.6 ± 5	0.5 ± 0.01
ZnO	65 ± 0.3	163 ± 5	76.5 ± 0.2	65.4 ± 1.6	99.6 ± 5	1.5 ± 0.10
ZnO/TiO_2_	75 ± 0.3	262 ± 5	26.9 ± 0.2	65.4 ± 2.6	95.3 ± 5	4.6 ± 0.5

In contrast, higher surface roughness causes an increase in membrane flux due to higher surface area^59–61^. The roughness parameters of the hybrid membrane improved gradually for TiO_2_ and ZnO nanocomposite membranes and decreased to 26.9 for ZnO/TiO_2_ nanocomposite membranes, indicating a smoother surface than the pure PVC membrane. The presence of NPs in the casting solution, which causes valley coverage on membrane surfaces, may help explain hybrid membranes' surface roughness. It is generally established that a smoother membrane surface can reduce foulant adherence at peaks and valleys as well as valley-to-peak penetration. Because of this, the hybrid membranes' antifouling capabilities outperformed those of the pure PVC membrane^62–64^.

**Figure 6:**
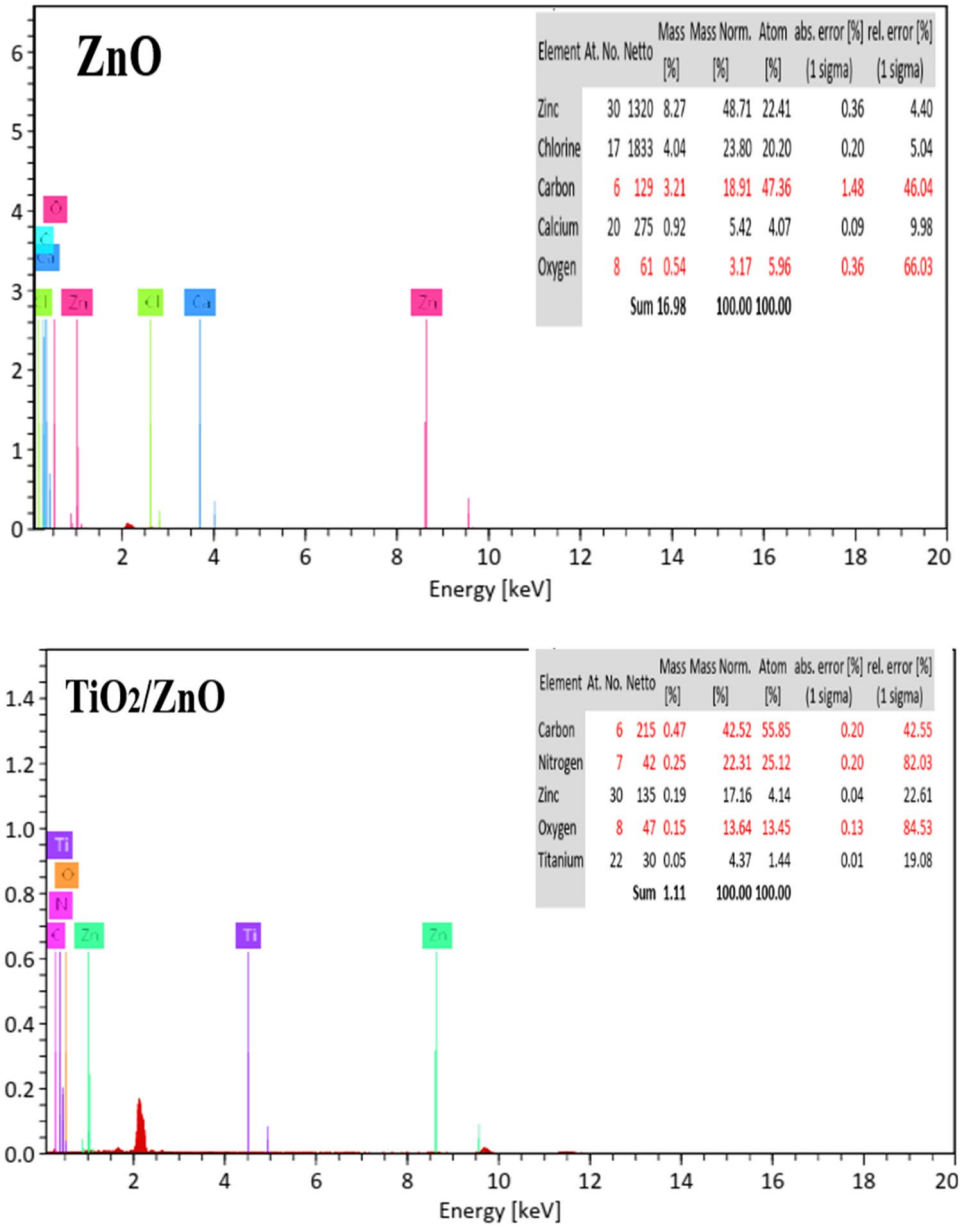
EDX analysis of ZnO, and TiO_2_/ZnO nanocomposite membranes.

**Elemental analysis** of the elemental compositions of ZnO, and TiO_2_/ZnO membranes and the relative amount of each element in the fabricated membranes which were obtained were confirmed through EDX analysis Fig. 6). As seen, The PVC membrane modified with ZnO NPs showed a (Zn) peak related to ZnO and for the PVC membrane modified with TiO_2_/ZnO nanocomposite both (Zn), and (Ti) peaks were observed due to both the ZnO, and TiO_2_, confirming the incorporation of the photocatalyst in the membrane. oxygen (O), carbon (C), chloride (Cl), and nitrogen (N) peaks also appeared associated with the composition of the PVC^65,66^.

#### FT-IR and Raman spectrum of the prepared membranes

**Figure 7:**
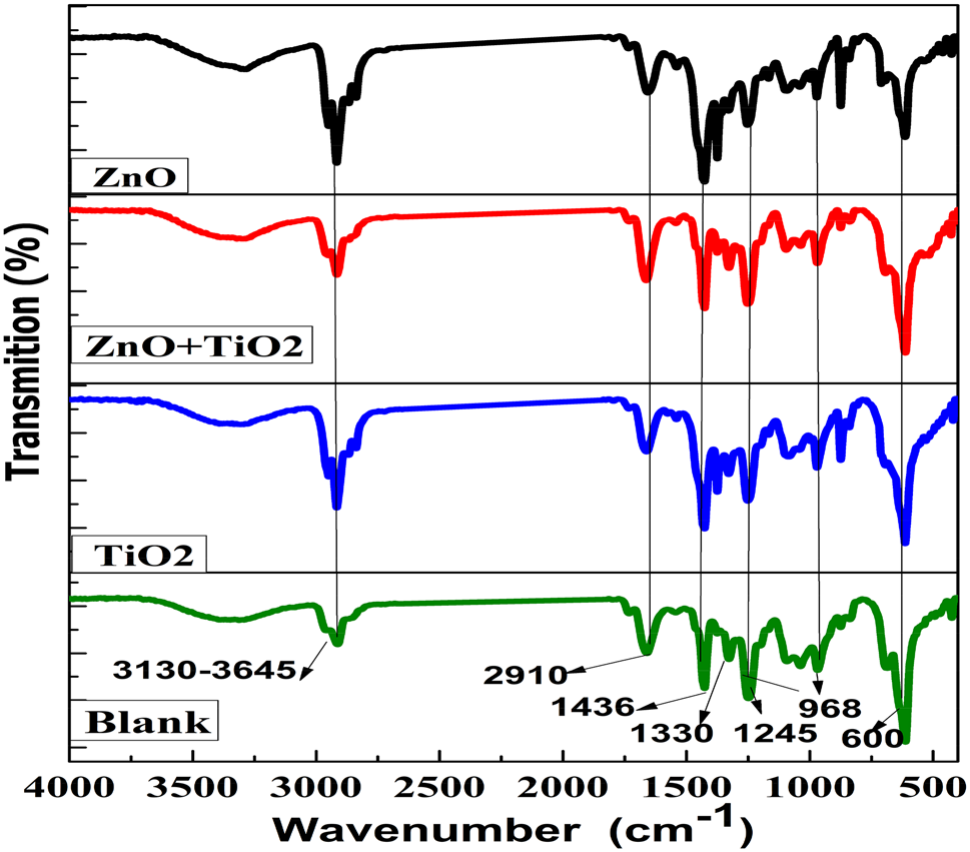
FTIR spectra for (blank, TiO_2_, ZnO, and ZnO/TiO_2_ nanocomposite membranes.

FT-IR was used to analyze the pure PVC and nanocomposite membranes' chemical structure (Fig. 7). The composite membranes exhibit all the characteristic peaks of pure PVC, indicating that the entrapped NPs did not disrupt the polymer structure. Specific bands and their assignments are summarized in Table 1. A typical stretching vibration of water hydroxyl (–OH) and carboxylic groups was detected in the spectrum of all membranes around 3130–3645 cm^−1^. C–H bond stretching vibrations are detected at 2910 cm^−1^. Peaks at 1436 cm^−1^ and 1667 cm^−1^ correspond to stretching the C=C and carbonyl (C=O) groups, respectively. The combined stretching vibration of C–H causes the absorbing band at 1330 cm^−1^. Furthermore, the peak at 600 cm^−1^ was caused by the stretching vibration of the C–Cl bond on the PVC. All the new peaks mentioned above were caused by the nucleophilic substitution of the PVC membrane, which resulted in some of the –Cl being present. Validated FTIR spectra of several hydrophilic groups may interact with polymer chains. The addition of NPs makes the dope solution more hydrophilic, improving the solvent/nonsolvent exchange rate during phase separation. In addition, 968 cm^−1^ and 1245 cm^−1^ absorbent band several bending vibrations inside and outside the C–H bonding plate^67–70^.

**Figure 8:**
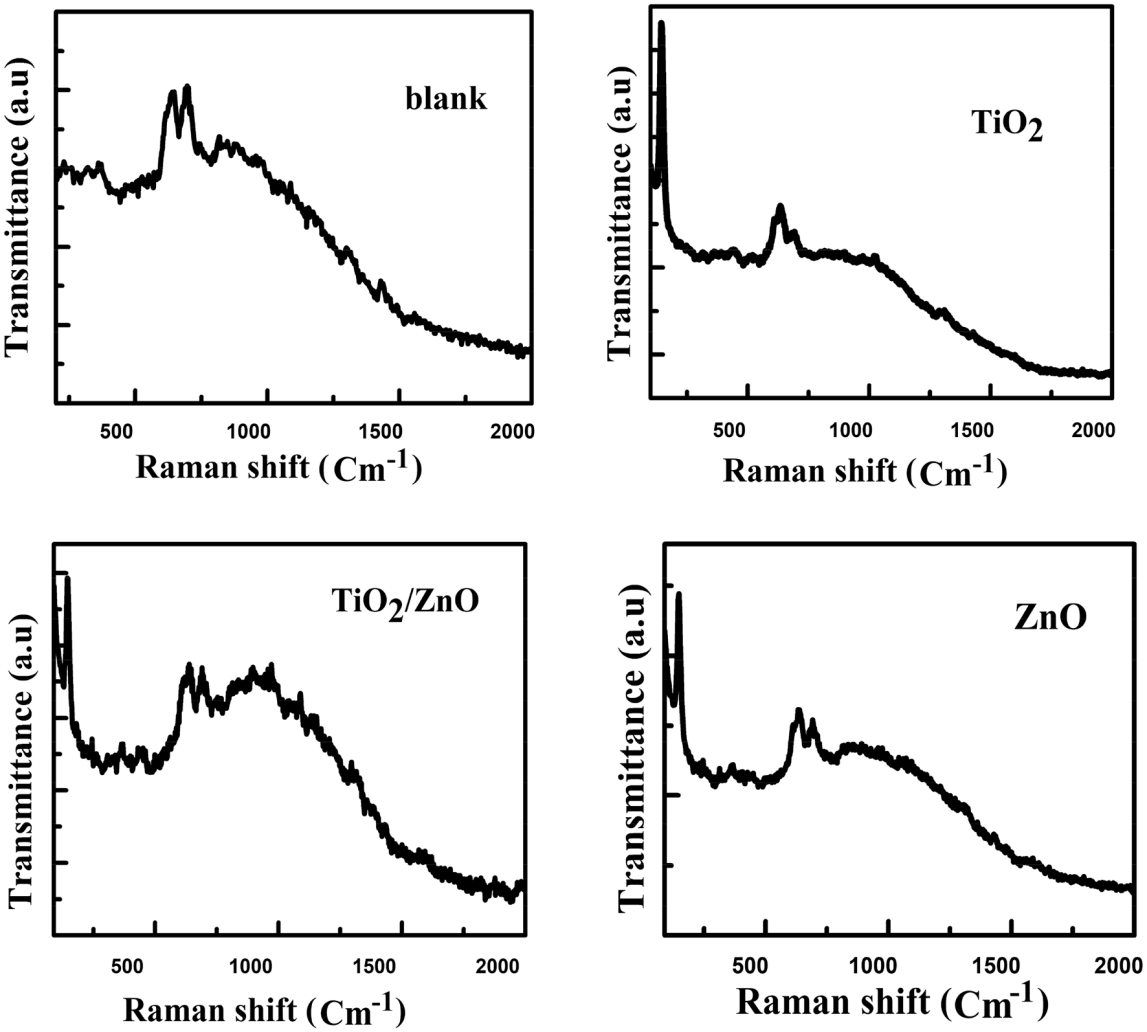
Raman spectra for (blank, TiO_2_, ZnO, and ZnO/TiO_2_ nanocomposite membranes.

All neat and nanocomposite membranes were analyzed using Raman spectroscopy (Fig. 8). Due to the C–Cl stretching vibration, two potent characteristic peaks for PVC can be observed at 636 and 693 cm^−1^^71–74^. According to factor group analysis, Anatase has six Raman active modes (A_1g_ + 2B_1g_ + 3E_g_). Ohsaka investigated the Raman spectrum of an anatase single crystal and concluded that the six allowed modes appear at 144 cm^−1^ (E_g_), 197 cm^−1^ (E_g_), 399 cm^−1^ (B_1g_), 513 cm^−1^ (A_1g_), 519 cm^−1^ (B_1g_), and 639 cm^−1^ (E_g_)^75–77^. A weak peak at 446 cm^−1^ can be attributed to rutile.

However, this indicates that the rutile phase content in the surface layer is low^78,79^. Among the observed peaks, the shift at 144 cm^−1^ is the strongest. This intense peak indicates that the TiO_2_ NPs in the membrane layer have some long-range order^80^. A new peak at 144 was observed in ZnO nanocomposite membranes related to ZnO NPs. The reduction in the intensity of the PVC characteristic peaks indicates that the chemical interaction between the functional groups of ZnO and TiO_2_ and the PVC matrix was successful. The composite membranes showed all the pure PVC characteristic peaks. Surprisingly, the addition of ZnO and TiO_2_ NPs altered the intensities of the PVC polymer peaks, confirming NP intercalation into the matrix.

#### The water contact angle and the membrane porosity

Table 1 shows the produced membranes’ total porosity data. The porosity measurements showed that the produced membranes all had good porosity in the range of (59–75%). This Table illustrates how the kind of NPs impacted the porosity of membranes. Thus, using NPs improves the membrane porosity's internal structure, which enhances the membrane characteristics and could lead to a rise in lateral flow rates through the membranes.

PVC membrane surfaces' hydrophilicity was assessed using the water contact angle. If the contact angle exceeds 90 degrees, the samples exhibit hydrophobic properties and demonstrate inadequate wetting. Nevertheless, in cases where the angle is below 90 degrees, the observed samples exhibit hydrophilic properties and demonstrate favorable wetting characteristics. These samples display a reduced contact angle with the NPs in comparison to the pure PVC sample. This observation substantiates the notion that the addition of nanoparticles (NPs) enhances the hydrophilicity of the samples, particularly in the context of water flux testing. The hydrophilicity of NPs was observed to increase with the number of nanoparticles, as indicated in Table 1^81^. Consequently, a decrease in the water contact angle indicates increased hydrophilicity. Without any modifications, the PVC membrane exhibited the highest water contact angle of 72.43. Conversely, the water contact angles of the PVC membranes modified with ZnO and ZnO/TiO_2_ were lower, suggesting an enhancement in the hydrophilicity of the membrane surface. Previous research has indicated that incorporating inorganic nanoparticles into polymeric membranes leads to a decrease in the contact angle^82–85^, and the same results have been reported in inorganic/PVC composite membranes^8,22^. Increasing membrane hydrophilicity generally improves fouling resistance because hydrophilic membranes absorb more water than hydrophobic membranes^86,87^.

#### Mechanical properties

**Figure 9:**
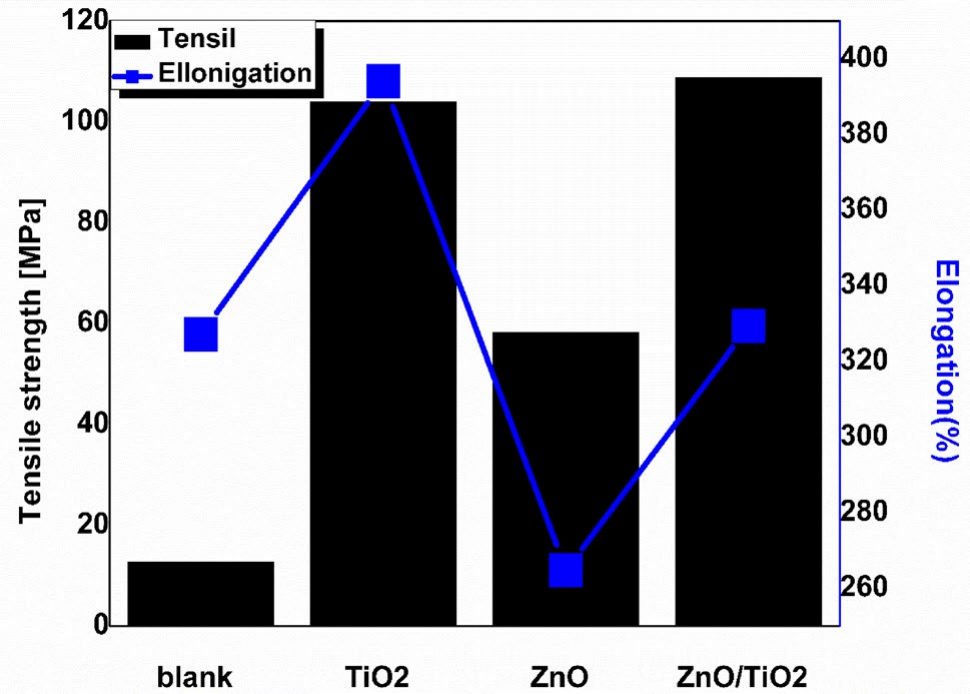
Tensile strength, and elongation of blank, TiO_2_, ZnO, and ZnO/TiO_2_ nanocomposite membranes.

The produced membranes had outstanding mechanical characteristics. The tensile strength of blank, TiO_2_, ZnO, and ZnO/TiO_2_ membranes is shown in Fig. 9. The TiO_2_/ZnO membrane had the highest tensile strength of the modified membranes compared to the unmodified PVC membrane. The results demonstrated that the addition of NPs to membranes increased their tensile strength even though adding NPs may cause the membrane to lose elasticity. This result is due to the placement of NPs in the membrane in the same direction and the alignment of ribbons perpendicular to the applied force. As a result of the reinforcement effect of inorganic NPs, the incorporation of inorganic NPs into composite membranes increases their mechanical strength. While the addition of NPs decreased the elongation at breakage. The high viscosity and coalescence action of the cast solution reduced elongation. Due to their large surface area and high aspect ratio, NPs interact well with PVC and reinforce PVC membranes. It can be concluded that the addition of NPs affects the membrane's mechanical properties^22^.

#### Membrane filtration performance

**Figure 10:**
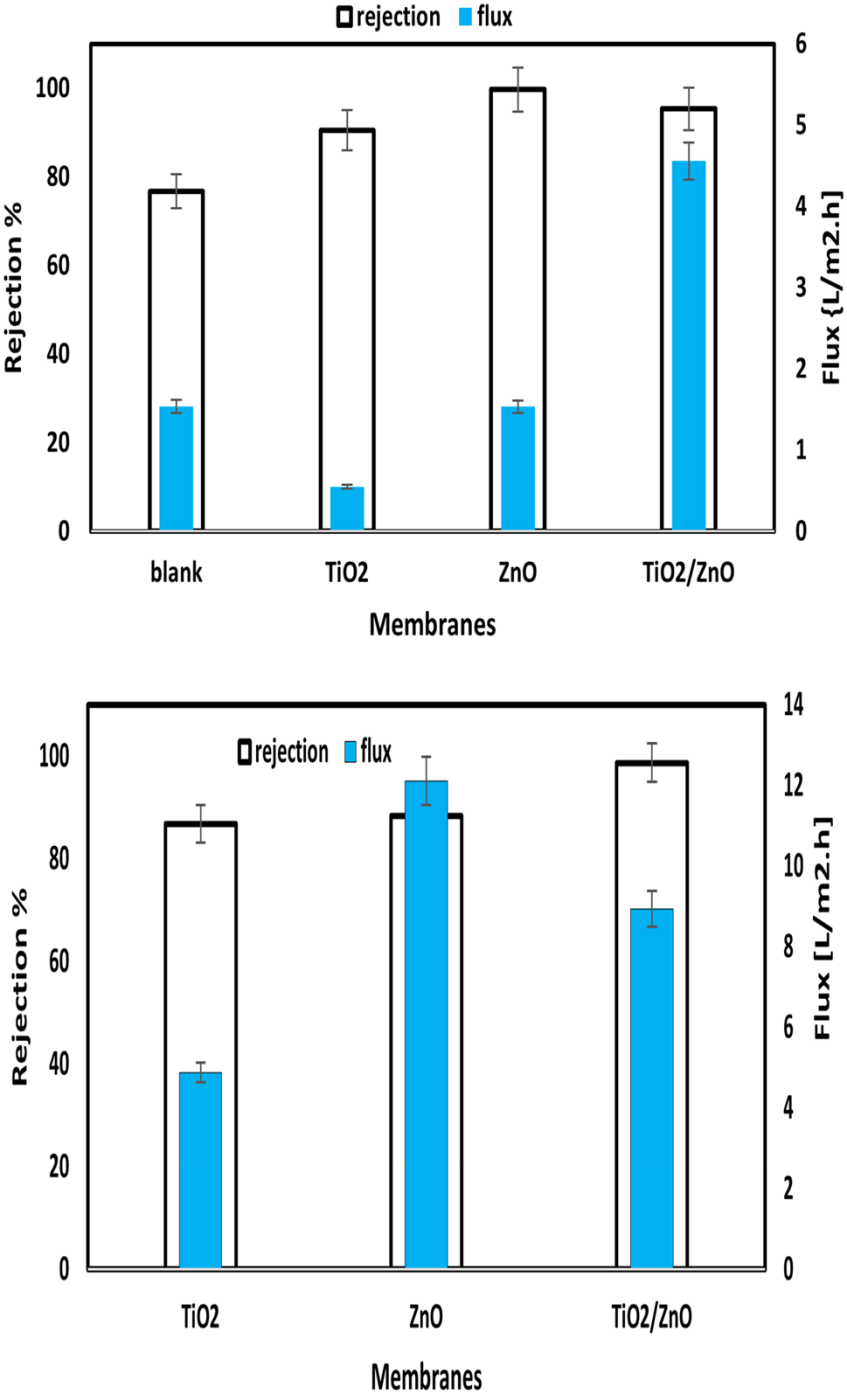
The rejection, and the pure water flux for blank, TiO_2_, ZnO, and ZnO/TiO_2_ nanocomposite membranes.

The effect of NPs on the rejection and the pure water flux was investigated after 28 min in the dark, as demonstrated in Fig. 10). Hence, the pure water flux of the membrane is 1.54 L/(m^2^ h) with an efficiency of 76.72%. The flux was decreased to 0.55 L/(m^2^ h) with the addition of TiO_2_ as a result of the largest roughness value, and the efficiency was enhanced to 90.56% for the ZnO membrane, but the water flux was improved to 1.53 L/(m^2^ h), and the efficiency increased to 98.90%. Nevertheless, after the embedded PVC membrane with ZnO/TiO2, the water flux and the efficiency were enhanced to 4.56 L/ (m^2^ h) and 95.31%, respectively. Several variables influence membranes' pure water flux, including hydrophilicity, thickness, surface pore radius, and porosity. As previously indicated, the presence of the hydrophilic inorganic NPs increases the water adsorption and wetting rate on the surface of nanocomposite membranes, allowing for easier water permeation through the membrane. In addition, the number of membranes and the mean pore radius both grow as NPs are incorporated into the PVC matrix. However, when all membranes were retested in sunlight 30 °C, and light irradiation was 250 KW h/m^2^, both efficiency and water flux increased.

Smaller particles can pass through the membrane structure because they are smaller than the pore size created. However, larger particles cannot pass through membranes because they are larger than the pore size created. In the case of photocatalytic membranes, however, the filtering mechanism that eliminates larger particles works in tandem with the photocatalytic destruction of contaminants. In one of our published studies, the mechanism of TiO_2_ NPs was described^46^. When exposed to visible light, excited state electrons captured oxygen and water to create the superoxidants O_2_· − and OH·, which degraded organic contaminants to produce carbon dioxide and water. When the photocatalyst is exposed to light, it produces hydroxyl radicals that destroy retained pollutants.

**Figure 11:**
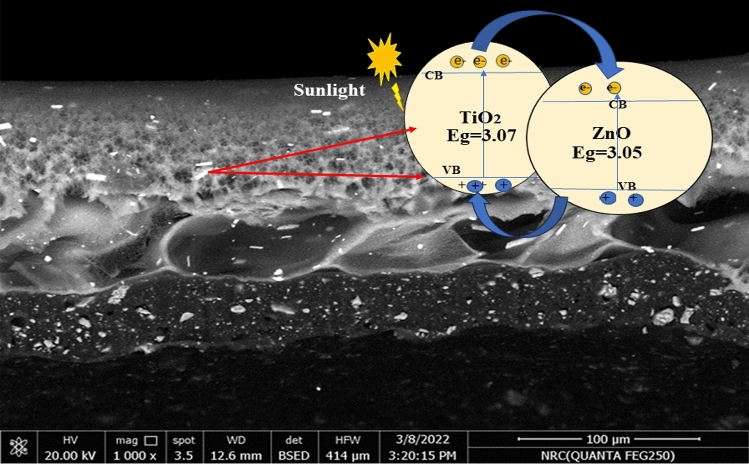
The excited electrons transfer mechanism inside the TiO_2_/ZnO membrane.

According to earlier research^14^, OH· and O_2_· − radicals are the main oxidizing species. Furthermore, the unoccupied electron–hole on the photocatalyst causes the pollutants to undergo simultaneous reduction and oxidation when they meet the membrane surface that has been doped with NPs. This prevents the formation of cake layers on the surface of the membrane, which may impede both photocatalytic activity and membrane permeability. The following reaction exemplifies the process of photocatalytic degradation of organic substances in organic wastewater, facilitated by the presence of a TiO_2_/ZnO nanocomposite and under the influence of solar illumination. Creation of the hydroxyl radical (OH·), which is the primary catalyst for the breakdown of organic materials, is created when the superoxide anion (O·_2_¯) is reduced by the electron in the conduction band CB (e¯_CB_), as seen in Process (Eqs. 10–16). Hydroxyl radicals (OH^•^) subsequently initiate the degradation of the organic pollutants present in the wastewater, resulting in their mineralization into CO_2_ and H_2_O. It has been proposed that the potent polarity hydroxyl radicals produced during the photocatalytic reaction could increase the flow by combining hydrogen bonds and van der Waals forces with water molecules. In contrast, the slight reduction in flow was attributed to mild membrane fouling, whereby humic acid molecules adhered to the surface and pores of the membrane, resulting in restricted pathways and impeding water permeation through the membrane, as demonstrated in Fig. 11). When the TiO_2_/ZnO nanocomposite is exposed to sunlight, the photoexcited electrons move between the C_B_ of TiO_2_ to the C_B_ levels of ZnO, and the holes move also between the V_B_ levels of TiO_2_ and ZnO NPs. Therefore, it is possible to improve the effectiveness of the interfacial charge migration to molecules that have been adsorbed to prolong the lifespan of the charge carriers.

10 $$\mathop {{\text{MO}}_{{2}} }\limits_{{\left( {{\text{Photoexcitation}}} \right)}} + {\text{h}}\upsilon \to {\text{MO}}_{{2}} \left( {{\text{e}}_{{{\text{CB}}}}^{ - } + {\text{h}}_{{{\text{VB}}}}^{ + } } \right)\quad {\text{; M}}:{\text{Ti}},{\text{Zn}}$$

11 $${\text{h}}_{{{\text{VB}}}}^{ + } + {\text{OH}}^{ - } \to {\text{OH}}^{ \cdot }$$

12 $${\text{e}}_{{{\text{CB}}}}^{ - } + {\text{O}}_{{2}} \to {\text{O}}_{2}^{ - \cdot }$$

13 $${\text{O}}_{2}^{ \cdot - } + {\text{H}}_{{2}} {\text{O}} \to {\text{OOH}}_{ + }^{ \cdot } {\text{OH}}^{ - }$$

14 $${\text{2OOH}}^{ \cdot } \to {\text{O}}_{{2}} + {\text{H}}_{{2}} {\text{O}}_{{2}}$$

15 $${\text{H}}_{{2}} {\text{O}}_{{2}} + {\text{O}}_{2}^{ - \cdot } \to {\text{OH}}^{ \cdot } + {\text{OH}}^{ - } + {\text{O}}_{{2}}$$

16 $${\text{OH}}^{ \cdot } + {\text{Organic}}\;{\text{pollutants}} \to {\text{CO}}_{{2}} + {\text{H}}_{{2}} {\text{O}}$$

e^−^·_CB_ and h^+^_VB_: are the electrons in the conduction band and the hole in the valence band, OH·: is the hydroxyl radical, O· _2_^−^ is a superoxide anion, OOH· is hydroperoxyl radical, and H_2_O_2_ is hydrogen peroxide.

**Figure 12:**
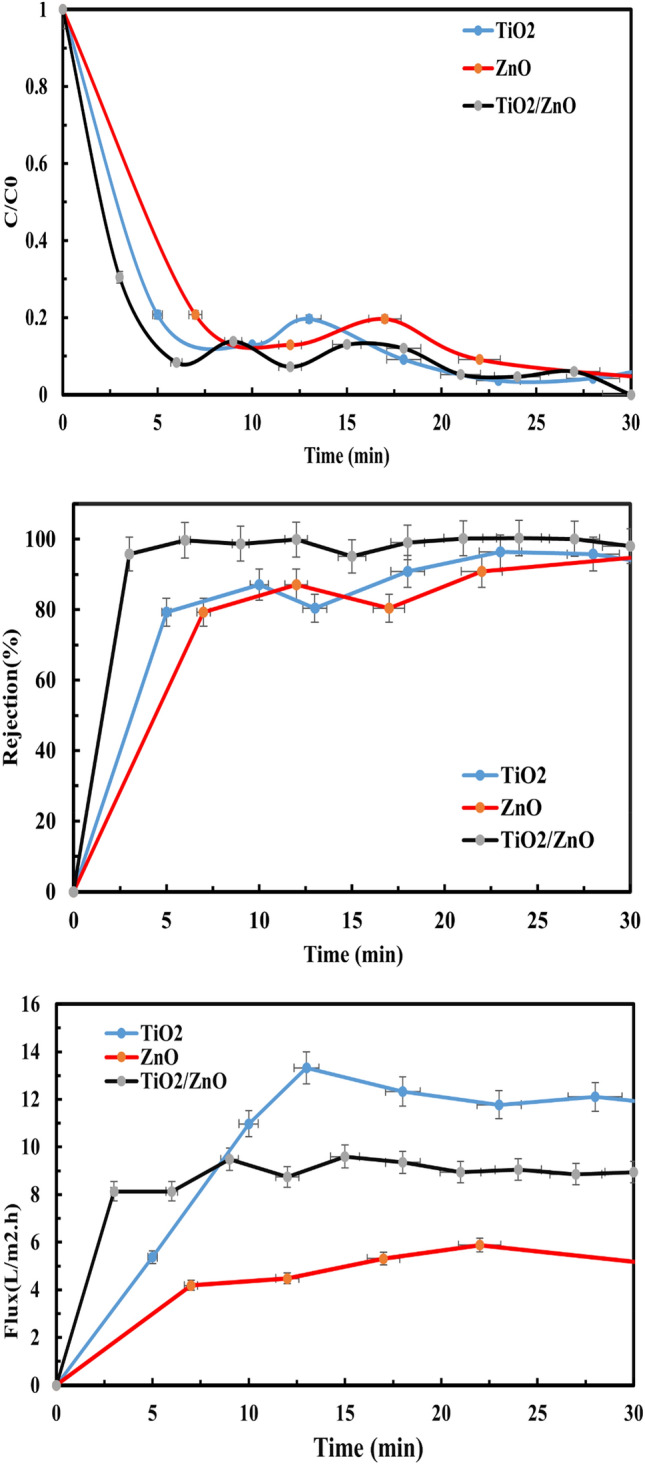
The photocatalytic activity, membrane removal efficiency, and flux in the presence of sunlight irradiation for (TiO_2_, ZnO, and TiO_2_/ZnO) photocatalytic membrane.

As shown in Fig. 11, membrane flux and removal increase as more contaminants are converted into smaller, harmless forms that can readily pass through the membrane. More research was done on how photocatalysis affects membrane permeability and removal properties. Figure 12 depicts all modified membranes' photocatalytic activity, membrane removal efficiency, and flux. Once ZnO was replaced with TiO_2_, overcoming the significant electron–hole recombination rate of TiO_2_/ZnO during the 28-min irradiation period enhanced the ability of ZnO ability to degrade pollutants. In addition, the flow was greater during photocatalytic purification compared to filtration alone. It has been suggested that the combination of water molecules through van der Waals forces and hydrogen bonds with highly polarized hydroxyl radicals produced by the photocatalytic process could increase the flow^88^.

Contrarily, the slight decrease in flux was caused by mild membrane fouling, in which humic acid molecules had adhered to the membrane's surface and pores, constricting pathways, and making it difficult for water to pass through the membrane^89,90^.

**Figure 13:**
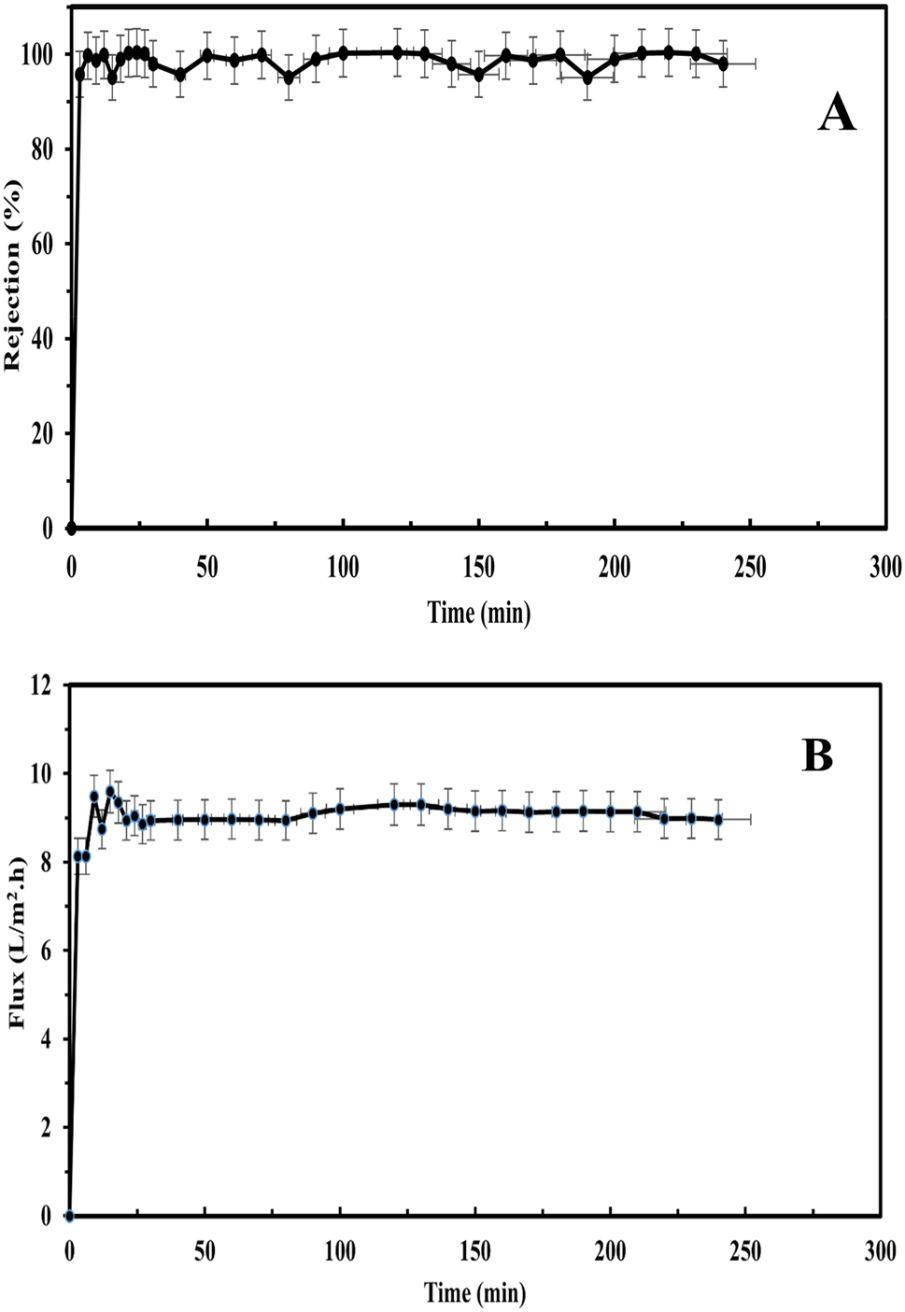
Photocatalytic performance. (**A**) Photocatalytic rejection of humic acid in synthetic surface water. (**B**) Water flux by TiO_2_/ZnO photocatalytic membrane.

Like experiments for the best membrane (TiO_2_/ZnO) in sunlight, an actual experiment of the best membrane (TiO_2_/ZnO) in sunlight, which was for a longer period of about 240 min, was also re-examined, studying both the rejection and the water flux, Fig. 13A and B).

As shown in Table 2, the overall performance of photocatalytic membranes supplemented with inorganic nanomaterial published in previous reports is compared with TiO_2_/ZnO PVC membrane in the work illustrated that TiO_2_/ ZnO PVC membrane possessed an excellent property, indicating clearly that this high-function modified membranes can expand the level of water treatment technique.

**Table 2:** Comparison performance of photocatalytic membranes supplemented with inorganic NPs published in previous studied.

Materials	Method	NPs	light	Flux L/m^2^ h	Efficiency	Pollutant	References
CeO_2_ on stainless steel	Facile spray coating	CeO_2_	UV lamp	–	99.96%	MB (10 ppm)	^91^
GO-TiO_2_ /(PAN)	layer-by-layer	GO-TiO_2_	UV 250 min	1.88	58.8%	(MB) 20 mg/L	^92^
N,Pd /TiO_2_ /PSf	Phase inversion	N,Pd co-doped TiO_2_	visible light 4 h	–	97%	Dye (eosin yellow) (100 mg/L)	^29^
N-TiO2/PDVF Hollow fiber	Dry/wet cospinning	N-doped TiO_2_	LED and UV 360 min	41.9	75% and 100% of visible and UV	Reactive Black 5 (RB5)	^93^
ZnIn_2_S_4_ /PVDF	Phase inversion	ZnIn_2_S_4_	halogen tungsten lamp 3 h	270	95%	Tetracycline 10 mg·L^−1^	^94^
NRGT/PS	Non-solvent-induced phase-separation	N-doped GO/TiO_2_ (NRGT)	UV, and sunlight150 min	210 and 220 with sun and UV	To 93% and 95% with sun and UV	(MB) 50 mg/L	^62^
TiO_2_@HNTs PVC	Phase inversion	TiO_2_@HNTs	UV	–	42.37%32.76 MB and RB	MB and RB dye 20 mg/L	^67^
(TNT)/PVDF	Phase inversion	Titania nanotubes	Hg lamps 90 min	–	45.50%	brilliant green (BG) dye	^95^
Au0.1Ag0.9/TiO_2_/CA	Phase inversion	Au0.1Ag0.9/TiO_2_	visible light	–	80%	pulp and paper factory (Chooka)	^96^
PVDF-TiO_2_ hollow fiber	Phase inversion	TiO_2_	UV A lamp 90–120 min	45	60%	Organic Compound COD	^97^
TiO_2_/ZnO/PVC	Phase inversion	TiO_2_/ZnO	Sunlight 28 min	9.3	99%	Humic acid (0.5 g/L)	Current study
TiO_2_/ZnO nanocomposites	Sol–gel and hydrotherma	TiO_2_/ZnO	xenon lamp 80 min	–	100%	Methylene blue (MB) (10 mg/L)	^98^
heterojunction ZnO/TiO_2_	Precipitation	ZnO/TiO_2_	UV 120 min	–	90%	RhB (10–5 M)	^99^
ZnO/TiO_2_ nanocomposite	Sol–gel	ZnO/TiO_2_	UV 3 h	–	96%	methylene blue 50 ppm	^100^
ZnO/TiO_2_ Thin film	Doctor blade	ZnO/TiO_2_	UV 12 h	–	74.04%	Acetaldehyde 150 ppm	^101^
C QDs modified ZnO/ TiO_2_ nanotube heterojunction	Electrospun–hydrothermal	C QDs ZnO/ TiO_2_	visible light 60 min	–	95%	Rh B (5 mg/L)	^102^
Cu–ZnO/TiO_2_ nanoparticles	Sol–gel	Cu–ZnO/TiO_2_	Visible light- 150 min	–	97%	Methyl orange	^103^
ZnO/TiO_2_	Sol–gel spin coating technique	ZnO/TiO_2_	UV 240 min	–	94%	Methylene blue (MB)	^104^

## Conclusion

Green TiO_2_ and ZnO NPs were successfully prepared using natural plant extract. A flat sheet of TiO_2_, ZnO, and TiO_2_/ZnO. A photocatalytic membrane was successfully fabricated using the phase inversion method to dope the photocatalyst. According to the hydrophilicity analysis, the PVC/ZnO membrane with a measured contact angle of 59.26° had the lowest contact angle. ZnO was able to increase the hydrophilicity of the membrane surface, according to the findings of the research. A macro-void has replaced the membrane's finger-like pore development. The hydrophilicity of the photocatalyst had less of an effect than the membrane surface's roughness and porosity. As the photocatalytic degradation rejection rate was successfully increased compared to the unmodified membrane, further photocatalytic investigation revealed that TiO_2_/ZnO was the tested membrane with the best performance. Under solar light irradiation, the membrane exhibited exceptional photodecomposition capability for removing humic acid from feed water, and the flow rate was increased. The integration of a green heterogeneous photocatalyst and membrane processing in our photocatalytic membrane enables the simultaneous execution of membrane separation and photodegradation. This integration offers several advantages, including a simplified system setup, economic benefits, and environmental sustainability. The significant intrinsic separation of humic acid and its robust photocatalytic activity are responsible for this phenomenon. Additional research on the loading of polyvinyl chloride (PVC) and the co-doping of other photocatalysts has the potential to finely adjust the performance, thereby enhancing the degradation of humic acid.

## Data Availability

The datasets generated during and/or analyzed during the current study are available from the corresponding author upon reasonable request.
